# Supplementary material for: Enzyme immunoassays (EIA) for serodiagnosis of human leptospirosis: specific IgG3/IgG1 isotyping may further inform diagnosis of acute disease
Source: PLoS Negl Trop Dis. 2022 Feb 23;16(2):e0010241. doi: 10.1371/journal.pntd.0010241 (PMC8901056; doi:10.1371/journal.pntd.0010241)
Supplement: S1 Table — LDA/HBO, Luanda/Huambo; HLA, Huila; AZ, Azores, LIS, Lisbon; ROC(AUC), receiver-operating characteristic (ROC) analysis with area under the curve (AUC). (DOCX) [file pntd.0010241.s003.docx]

**S1 Table.** Diagnostic performance of other Leptospira extracts


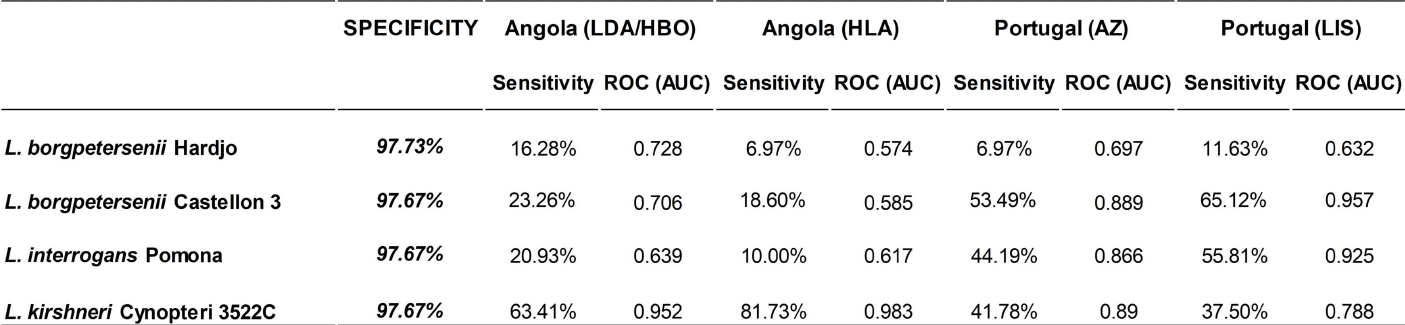


Legend: LDA/HBO, Luanda/Huambo; HLA, Huila; AZ, Azores, LIS, Lisbon; ROC(AUC), receiver-operating characteristic (ROC) analysis with area under the curve (AUC).
