# Supplementary material for: Enzyme immunoassays (EIA) for serodiagnosis of human leptospirosis: specific IgG3/IgG1 isotyping may further inform diagnosis of acute disease
Source: PLoS Negl Trop Dis. 2022 Feb 23;16(2):e0010241. doi: 10.1371/journal.pntd.0010241 (PMC8901056; doi:10.1371/journal.pntd.0010241)
Supplement: S1 Text — (DOCX) [file pntd.0010241.s005.docx]

**S1 Text**

**Step-by-step EIA protocol for detection of anti-leptospira antibody in human serum**

**1. Production of heat-killed Leptospira antigen**

1. Centrifuge a 5 mL culture of Leptospira (10^8^/mL) at 12,000g for 10 min and discard the supernatant.
2. Wash the pellet with 5 mL PBS, centrifuge at 12,000 g.
3. Count the cells under a Dark Field Microscope (DFM). Adjust to 10^7^-10^9^ cells/mL.

*Note 1: if DFM is not available, Leptospira can be quantified by qPCR.*

1. Aliquot 1 mL of bacteria into 2 mL tubes.
2. Subject up to 5 rounds of freeze-thawing, followed by 2-3 rounds of heat killing for 15 min at 95°C. Heat killing is confirmed by checking under the DFM: no motile Leptospira are expected to be visible.
3. Determine the protein concentration using the Modified Lowry Protein Assay Kit according to the manufacturer’s protocol.

**2. Quantification of Leptospira-specific antibody (IgM and IgG) in serum by EIA**

1. Coat a plate with antigen: dilute heat-killed Leptospira in 1X coating buffer (sodium carbonate, pH8.4-9.6) to a final concentration of 1x10^5^-10^8^/well or 100 μl/well of 4 mg/mL of heat killed Leptospira in a 96 well plate. Cover the plate and incubate overnight at 4^o^C.
2. Next morning, wash the plate using the EIA plate washer and run a total of 4 washes with 300 μL Wash Buffer.
3. To block the plate: pour blocking buffer into a new reagent reservoir and add 250 μL of blocking buffer (1XPBST+1%BSA-prepare fresh) in each well (using a multichannel pipet) and incubate for 1-2h at room temperature (RT) or 37^o^C.

*Note 2: If a plate washer is not available use a multi channel pipet to dispense 300* μ*l of wash buffer per well, dump the wash buffer into a sink and tap dry over a layer of 3-4 clean tissue papers.*

*Note 3: at this point, the plate can be stored @4^o^C for maximum of 2 days (cover the plate or put plate inside a ziplock bag) or continue following the protocol.*

1. Wash the plate two times with 300 μL Wash Buffer. Tap dry over a layer of 3-4 clean tissue papers.
2. Dilute the serum samples (ex 1:100) in blocking buffer and add 100 μL of diluted serum sample into each well of the Test plate, cover the plate and incubate for 1.5 hr at RT or 1h at 37^o^C.
3. Wash the Test plate 4 times; tap dry over a layer of 3-4 clean tissue papers.
4. Clean the plate washer: Dip the wash head probe in 1XPBST in the blue boat provided with the instrument and run a Wash plate twice to remove any primary sera sticking onto the wash probe head (see Note 2).
5. Dilute the secondary antibody-HRP (IgM, IgG, IgA) in blocking buffer and pour this into a new reagent reservoir; add 100 μl to the wells of the Test plate using a multichannel pipet; cover the plate and incubate at 1 hr at RT 37^o^C for 30 min.
6. Wash the Test plate 4 times on the plate washer; tap dry over a layer of 3-4 clean tissue papers.
7. Clean the plate washer: dip the wash head probe in 1XPBST in the blue boat provided with the instrument and run a Wash plate with the “2 wash” program to remove any HRP-conjugated antibody sticking onto the wash probe head.

*Note 4: If buffer is left in the instrument solutes form deposits and block the tubing.*

1. Add 100 μl of TMB SureBlue substrate at room temperature into each well of the Test plate using a multichannel pipet.
2. Cover the plate and incubate for 15 minutes at RT or 37^o^C.
3. Add 100 μL of TMB Stop Solution into each well and read the absorbance @450nm in the SpectraMax EIA reader; save the file and export the raw data as “.xls” file for analysis.
4. Connect the washer to the MilliQ bottle and run a wash cycle with MilliQ.

*Note 5. We use two 96-Well plates to run the enzymatic reaction: one is labeled Test Plate and contains the serum samples subject to the study and the second plate is a Wash plate used to clean up the plate washer instrument after the washes.*

*Note 6. Before starting the enzymatic reaction part of this protocol aliquot the volume of TMB SureBlue required for the EIA in a 50 mL tube and keep it at RT covered in foil (TMB substrate is light sensitive and is to be used at RT).*

*Note 7. This protocol can be modified to test antibody levels against recombinant proteins as antigens. Instead of heat-killed Leptospira coat the plates with 0.1 – 1 μg/mL of purified recombinant protein in coating buffer in step 1 and follow the protocol from step 2.*
