# Supplementary material for: Enzyme immunoassays (EIA) for serodiagnosis of human leptospirosis: specific IgG3/IgG1 isotyping may further inform diagnosis of acute disease
Source: PLoS Negl Trop Dis. 2022 Feb 23;16(2):e0010241. doi: 10.1371/journal.pntd.0010241 (PMC8901056; doi:10.1371/journal.pntd.0010241)
Supplement: Attachment — Submitted filename: Comments.docx [file pntd.0010241.s006.docx]

The paper reported affordable in-house enzyme immunoassays using Leptospira serovars whole protein extracts and recombinant proteins, individually and in combination. The test can be performed for laboratory confirmation of leptospirosis to replace the laborious microscopic agglutination test (MAT). Interestingly, the authors showed increased sensitivities when a combination of antigens were used. This approach has been used previously to increase diagnostic sensitivity in many diseases. In general, the paper addresses an important issue, since the gold standard test (MAT) has several limitations. However, there are some concerns which should be considered.

- The study lake sera of healthy individual from same endemic areas/countries. These sera are better to assess specificity of the test.
- Purity of the recombinant proteins and their molecular sizes are not shown in Figures.
- It is not clear why the authors used sera of infected mice.
- In line 30: put the acronym (AO) in full when come first.
- Fig 1 could be omitted.
- In line 261: it is stated that whole cell extract from eight Leptospira serovars were used, however, fig 5, table 2 and table S1 show only results of 4 serovars. Some results are probably missing!
- In EIA protocol, please include Ph of the coating buffer (sod carbonate) and content/nature of the washing buffer.
- Line 718: please indicate whether substrate was incubated at dark?
- For figs 3-6 and S1, it is not clear what do the horizontal lines represent?
