# Supplementary material for: Enzyme immunoassays (EIA) for serodiagnosis of human leptospirosis: specific IgG3/IgG1 isotyping may further inform diagnosis of acute disease
Source: PLoS Negl Trop Dis. 2022 Feb 23;16(2):e0010241. doi: 10.1371/journal.pntd.0010241 (PMC8901056; doi:10.1371/journal.pntd.0010241)
Supplement: Attachment — Submitted filename: Response to reviewers 020122.docx [file pntd.0010241.s007.docx]

**Response to the Editor and to the Reviewers**

We appreciate the comments made by the editor and the reviewers and addressed all issues raised in this revision of the manuscript. Our answers are highlighted in blue.

**Editor Comments**
 
One significant point raised in the reviews is the age of the sera used, and how the collection and storage conditions might affect the results. Please address this point in the text by either performing some tests of your ELISA-based diagnostics with fresh sera or by acknowledging the possible limitations of using sera collected years ago.

Loss of quality of Ig in serum stored over long periods of time is usually a problem for IgM. We tested IgG in our study. Loss of quality of the serum would be reflected in loss of sensitivity of the assay which was not observed (100% sensitivity using a combination of Leptospira extracts). We added the following to the methods: Serum samples were collected between 2009 and 2015 and were kept at -20ºC without glycerol until use in 2018/2019.

**Reviewers Comments**

Reviewer #1: The paper reported affordable in-house enzyme immunoassays using Leptospira serovars whole protein extracts and recombinant proteins, individually and in combination. The test can be performed for laboratory confirmation of leptospirosis to replace the laborious microscopic agglutination test (MAT). Interestingly, the authors showed increased sensitivities when a combination of antigens were used. This approach has been used previously to increase diagnostic sensitivity in many diseases. In general, the paper addresses an important issue, since the gold standard test (MAT) has several limitations. However, there are some concerns which should be considered. 
•       The study lake sera of healthy individual from same endemic areas/countries. These sera are better to assess specificity of the test.

We agree it would have been better to use sera from an endemic area (Angola and Portugal) but we did not have access to those samples. Thus, we used sera from a non-endemic area. This has been clarified in methods and a paragraph added to the discussion (line 414).

•       Purity of the recombinant proteins and their molecular sizes are not shown in Figures.

Line 185. Examples of purified rLigA_7-13_, rLigB_1-6_ and rLipL32 are provided in new Fig S1.

•       It is not clear why the authors used sera of infected mice.

Line 241: To confirm the potential sensitivity of the three leads in comparison with other candidates, we tested purified rLigA, rLigB, rLipL32, rTolC and rFliE using serum from C3H-HeJ mice infected with *L. interrogans* sv Copenhageni FioCruz. We confirmed that unlike TolC and FliE, the three recombinant proteins LigA, LigB and LipL32 were highly immunogenic in mice (**Fig S2**).

•       In line 30: put the acronym (AO) in full when come first.

Corrected. 
•       Fig 1 could be omitted.

We deleted the former fig1 and re-numbered the others.
•       In line 261: it is stated that whole cell extract from eight Leptospira serovars were used, however, fig 5, table 2 and table S1 show only results of 4 serovars. Some results are probably missing!

Table 2 (4 serovars) and Table S1 (4 serovars) contain the 8 described serovars.
•       In EIA protocol, please include Ph of the coating buffer (sod carbonate) and content/nature of the washing buffer.

pH 8.4-9.6 was included in the protocol
•       Line 718: please indicate whether substrate was incubated at dark?

We don’t incubate in the dark.
•       For figs 3-6 and S1, it is not clear what do the horizontal lines represent?
The horizontal lines represent the ELISA cutoff.

Reviewer #2: Fortes-Gabriel et al, describe an Enzyme immunoassays (EIA) for serodiagnosis of human leptospirosis and describe that IgG3/IgG1 can help define acute disease. This is a great effort to improve human serological diagnosis in Leptospirosis as an alternative to the laborious and expensive MAT for screening acute infections in areas where circulating serovars of pathogenic Leptospira are well defined.

Although the article is clearly described, there is room for improvements such as the following:

1.      The serum collection is old (2010 and therefore the quality of these sera can be affected by the preservation methods. The text does not indicate whether they were preserved at -20 ° C or -80 ° C with or without glycerol. This maintenance directly affects the quality and preservation of the immunoglobulins, which can give variable results. This should be included in the methodology and discussed in the results.

Loss of quality of Ig in serum stored over long periods of time is usually a problem for IgM. We tested IgG in our study. Loss of quality of the serum would be reflected in loss of sensitivity of the assay which was not observed given that we detected positive cases with 100% sensitivity using a combination of Leptospira extracts.

We added the following to the methods line 123: Serum samples were collected between 2009 and 2015 and were kept at -20ºC without glycerol until use in 2018/2019.

2.      Date of collection of the serum are not mentioned for PT (AZ), PT (LIS) and healthy patients from Florida
We included the dates.

3.      Line 61-62. Brucellosis is another zoonotic disease common as differential diagnosis in the African continent. Please consider this differential to be include it.

We included brucellosis.

4.      It is recommended to include animal reservoirs in the countries that participate and sources of contagion in the description of the disease so the One Health concept is fully addressed.

We included this concept in line 79.

5.      Could this immunoassay be adapted and be useful in animals in these regions? this can be included in the discussion

We believe this immunoassay could be adapted to be used for seroprevalence in animals in endemic areas. That is the reason we provide an extensive protocol in Supplemental Material and submitted this to an Open Source journal so people all over the world can adapt, test and use as applicable. We added a line on this in discussion.

**Production Comments**

Figure Files:
While revising your submission, please upload your figure files to the Preflight Analysis and Conversion Engine (PACE) digital diagnostic tool, <https://pacev2.apexcovantage.com>. PACE helps ensure that figures meet PLOS requirements. To use PACE, you must first register as a user. Then, login and navigate to the UPLOAD tab, where you will find detailed instructions on how to use the tool. If you encounter any issues or have any questions when using PACE, please email us at [figures@plos.org](mailto:figures@plos.org).
Figures have been checked using PACE.

Data Requirements:
Please note that, as a condition of publication, PLOS' data policy requires that you make available all data used to draw the conclusions outlined in your manuscript. Data must be deposited in an appropriate repository, included within the body of the manuscript, or uploaded as supporting information. This includes all numerical values that were used to generate graphs, histograms etc.. For an example see here: <http://www.plosbiology.org/article/info%3Adoi%2F10.1371%2Fjournal.pbio.1001908#s5>.
The data is included in Supplemental Material.

Reproducibility:
To enhance the reproducibility of your results, we recommend that you deposit your laboratory protocols in protocols.io, where a protocol can be assigned its own identifier (DOI) such that it can be cited independently in the future. Additionally, PLOS ONE offers an option to publish peer-reviewed clinical study protocols. Read more information on sharing protocols at <https://plos.org/protocols?utm_medium=editorial-email&utm_source=authorletters&utm_campaign=protocols>
The protocols included in this paper are extensive to ensure reproducibility.

References
Please review your reference list to ensure that it is complete and correct. If you have cited papers that have been retracted, please include the rationale for doing so in the manuscript text, or remove these references and replace them with relevant current references. Any changes to the reference list should be mentioned in the rebuttal letter that accompanies your revised manuscript. If you need to cite a retracted article, indicate the article's retracted status in the References list and also include a citation and full reference for the retraction notice.
Reference list was checked and there are no retracted papers.
